# Epidermolysis Bullosa Acquisita: The 2019 Update

**DOI:** 10.3389/fmed.2018.00362

**Published:** 2019-01-10

**Authors:** Hiroshi Koga, Catherine Prost-Squarcioni, Hiroaki Iwata, Marcel F. Jonkman, Ralf J. Ludwig, Katja Bieber

**Affiliations:** ^1^Department of Dermatology, Kurume University School of Medicine, and Kurume University Institute of Cutaneous Cell Biology, Fukuoka, Japan; ^2^Department of Dermatology, APHP, Avicenne Hospital, Referral Center for Autoimmune Bullous Diseases, Bobigny, France; ^3^Department of Dermatology, Hokkaido University Graduate School of Medicine, Sapporo, Japan; ^4^Department of Dermatology, Center for Blistering Diseases, University Medical Center Groningen, University of Groningen, Groningen, Netherlands; ^5^Lübeck Institute of Experimental Dermatology, University of Lübeck, Lübeck, Germany

**Keywords:** epidermolysis bullosa acquisita, animal models, diagnosis, treatment, pathogenesis

## Abstract

Epidermolysis bullosa acquisita (EBA) is an orphan autoimmune disease. Patients with EBA suffer from chronic inflammation as well as blistering and scarring of the skin and mucous membranes. Current treatment options rely on non-specific immunosuppression, which in many cases, does not lead to a remission of treatment. Hence, novel treatment options are urgently needed for the care of EBA patients. During the past decade, decisive clinical observations, and frequent use of pre-clinical model systems have tremendously increased our understanding of EBA pathogenesis. Herein, we review all of the aspects of EBA, starting with a detailed description of epidemiology, clinical presentation, diagnosis, and current treatment options. Of note, pattern analysis via direct immunofluorescence microscopy of a perilesional skin lesion and novel serological test systems have significantly facilitated diagnosis of the disease. Next, a state-of the art review of the current understanding of EBA pathogenesis, emerging treatments and future perspectives is provided. Based on pre-clinical model systems, cytokines and kinases are among the most promising therapeutic targets, whereas high doses of IgG (IVIG) and the anti-CD20 antibody rituximab are among the most promising “established” EBA therapeutics. We also aim to raise awareness of EBA, as well as initiate basic and clinical research in this field, to further improve the already improved but still unsatisfactory conditions for those diagnosed with this condition.

## Epidemiology

The incidence of most autoimmune blistering diseases is increasing. Although the incidence of epidermolysis bullosa acquisita (EBA) is not known in detail, it is estimated to be rare. The most common autoimmune subepidermal blistering disease, bullous pemphigoid (BP), is reported to have an annual estimated incidence between 2.4 and 21.7 per million (1, 2). By contrast, the estimated incidence of EBA is reported to be <0.5 per million (3–7). In South Korea, the incidence and prevalence of EBA is estimated to be higher than that of previous reports (8), but the exact epidemiologic data have not been surveyed. In Germany, the EBA prevalence has recently been determined to be 2.8 cases per million (9). This ethnical difference may be due to the reported association of EBA with the human leukocyte antigen (HLA) class II (10–12). EBA occurs at any age; the onset age in previous case reports exhibit a wide range from 1 to 94 years old (13–15). Two onset age peaks are reported; the second and seventh decades (9).

## Clinical Presentation

**Figure 1:**
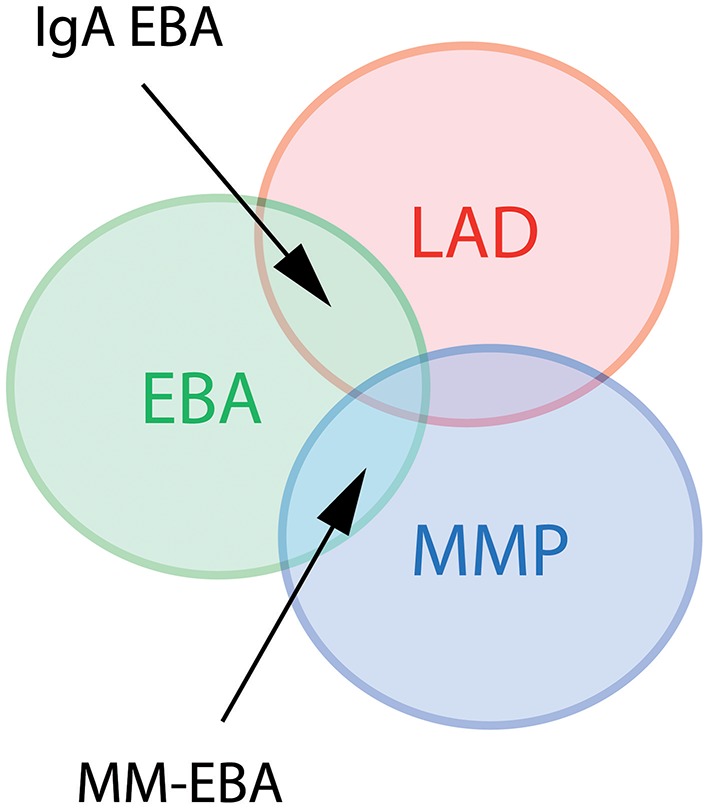
Diagram of different clinical forms of EBA. LAD, linear IgA disease. MMP, mucous membrane pemphigoid; EBA, epidermolysis bullosa acquisita; MM-EBA, mucous membrane EBA.

Several clinical EBA manifestations have been described: (i) the classical/mechano-bullous form and (ii) the non-classical/non-mechano-bullous forms (16). The latter includes BP-like EBA that meets the clinical criteria of both EBA and BP, mucous membrane (MM)-EBA that is clinically defined by predominant mucous membrane lesions, IgA-EBA that is defined by the IgA class of immune deposits, and Brunsting-Perry-like EBA (Figure 1). Few patients may have a MM-IgA-EBA. The relative frequencies of these different clinical forms of EBA reported in the few series in the literature (17–21) depend on the morphological and/or serological diagnostic means available to the authors (Table 1). The two most common presentations of EBA are the classical/mechano-bullous and the BP-like forms.

**Table 1:** Clinical variants of EBA in the series of the literature.

**References**	**Years of study**	***n***	**Classica/mechano-bullous**	**Brunsting-Perry like**	**Bullous pemphigoid like**	**Mucous membrane EBA**	**IgA EBA**
Briggaman (17)	<1985	12	4 (30%)	0	5 (40%)	1 (8%)	2 (17%)
Kim (18)	1994–2009	30	11 (36.7%)	2 (6.7%)	14 (46.7%)	2 (6.7%)	1 (3.3%)
Buijsrogge (19)	2002–2008	38	13 (34%)	1 (2.6%)	13 (34%)	2 (5.2%)	9 (24%)
Iranzo (20)	1985–2012	12	5 (42%)	1 (8.3%)		4^a^ (33%)	
Seta (21)	1983–2013	77	42 (56%)	1 (1.2%)	21^b^ (27%)	11^c^ (14%)	2 (2.4%)

a 2 patients had the mixed form,

b 1 had a prurigo-like form,

c *11 had mucous membrane-EBA, including 2 with isolated IgA deposits*.

It should be recognized that in an individual EBA patient, clinical presentation may change over time. Notably, patients may switch from a BP-like form to a classical/mechanobullous form or when mucous membrane lesions appear secondarily from a BP-like form to a MM-EBA (22).

**Figure 2:**
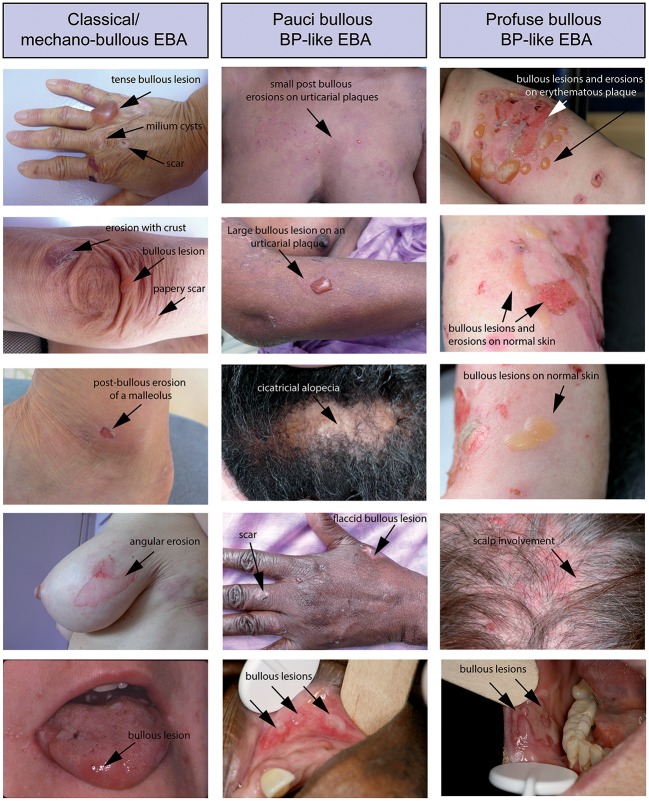
Different clinical forms of EBA. All patients were documented at the first visit in a center for auto-immune bullous disease. **(Left)** Patient with a classical/mechanobullous form of EBA: lesions are preferably localized to the extensor skin surfaces and trauma-prone sites, i.e., dorsal hands knees elbows and ankles. Tense or flaccid bullous lesions are surrounded by non-inflamed skin; erosions are covered or not by crusts; one erosion with angular contours had been induced by adhesive plaster; old lesions have healed with milium formation and/or are atrophic papery scar. **(Middle)** Patient with a BP-like form of EBA, with little blistering: urticarial plaques with small or large bullous lesions as in BP, but location of lesions on extensor areas of limbs, hands and scalp, and scars and extensor areas of the face (not shown) and limbs (atypical for BP). **(Right)** Patient with a BP-like form of EBA, with extensive blistering: bullous lesions and erosions on erythematous skin in flexural areas of limbs (tight and arm) as in BP but also bullous lesions and erosions on normal skin and involvement of extensor area of the limbs and scalp, atypical for a BP involvement of the scalp (not shown) and both flexural and extensor areas of limbs extremities with bullous lesions and erosions on erythematous but also normal skin. The tongue and the lips are the most frequent sites of mucosal lesions in all EBA variants. Other mucosal lesions (not shown) are possible regardless of the variant of EBA The involvement of nasal and buccal mucous membrane are visible in all EBA variants.

Regardless of the clinical form, patients present with cutaneous-mucous fragility, which is easily suspected when the lesions are on trauma-prone areas. Bullous lesions or erosions that are linear or with angular contours can also provide evidence of this fragility (Figure 2). Questioning the patient can confirm that bullous lesions appear immediately or a few hours after a trauma which can be minimal. This fragility can be quantified by applying an analogical visual scale. Because in EBA the subepidermal cleavage is deep on the dermal side of the basement membrane zone (BMZ), the cutaneous blisters can persist for a long time, collapsing and becoming flaccid before their rupture; they can also be haemorrhagic.

## Classical/Mechano-Bullous EBA

Two cases of an adult-onset, acquired blistering disease resembling patients with hereditary dystrophic epidermolysis bullosa were reported by Elliott (23). Other similar cases were described in early decades of the twentieth century (24, 25). The first to actually coin the term “epidermolysis bullosa acquisita” was probably Hundley and Smith (26). A landmark paper by Roenigk and colleagues was published in 1971, who described three new cases of EBA, reviewed the world literature and proposed the first diagnostic criteria for classical/mechano-bullous EBA (27).

The criteria of Roenigk were modified once immunological tests for EBA diagnosis had been developed. Advanced clinical criteria of the classical/mechanobullous form have been published in 2017 (16). Current clinical diagnostic criteria for the classical/mechanobullous form are skin fragility, blisters, or erosions on non-inflamed or scarred skin, scarring and milium formation, preferably located at trauma-prone sites and the extensor skin surface (dorsal hands, elbows, knees, Achilles tendon, feet) with possible nail dystrophy and scarring alopecia (Figure 2). Fibrosis of the hands and fingers leading to a mitten-like deformity may occur in patients with severe disease reminiscent of hereditary dystrophic epidermolysis bullosa. Mucosal involvement may also occur, but it is not predominant.

## BP-like EBA

BP-like form of EBA was first described by Gammon et al. (28). This occurred shortly after the demonstration by Nieboer et al. (29) and Yaoita et al. (30) that EBA autoantibodies are deposited in the anchor fibrils zone, which allowed for a definite diagnosis by immunoelectron microscopy (IEM), even though the clinic was (based on the criteria by Roenigk) atypical. Indeed, patients with a BP-like form of EBA have generally profuse skin lesions suggestive of a BP in some areas and an EBA in others (17, 31, 32). The patients have pruritus, tense bullae and erosions on inflamed erythematous or urticarial skin as well as trauma-induced bullous lesions surrounded by normal skin (Figure 2). The lesions are on the trunk and folds but also on limb extensor areas and distal extremities. The face can be affected. Mucosal involvement is also possible, but it is not predominant. Finally, lesions heal most often leaving atrophic scars and milia cysts as in the mechanobullous form.

## MM-EBA

The high frequency of mucosal lesions in EBA, in particular tongue and lip involvement, was highlighted by Dahl (33). Currently, MM-EBA cases are defined as EBA that mainly affects mucous membranes with a squamous epithelium (16, 33–35), such as the mucous membrane of the mouth, pharynx, esophagus, epiglottis, conjunctiva, genitalia, anus, and respiratory tract in malpighian metaplasia, especially the trachea and bronchi. Only one of these sites can be involved and remain so for a long time before a second localization appears in the case of inadvertent discontinuation of the treatment, a decrease in dose or no treatment (36). These cases are frequently misdiagnosed.

As on the skin, bullous lesions of mucous membranes rupture late in MM-EBA. Thus, intact blisters are frequently seen on mucous membranes in comparison to mucous membrane pemphigoid (MMP), in which they are rare (Figure 2). The erosions on the mucous membranes are similar in MM-EBA and classical MMPs, except in the esophagus where they can be linear, caused by mucous membrane fragility and the passage of the fibroscope (37). The cicatricial lesions (atrophic scars, synechiae, and stenosis) are identical in MM-EBA and MMP. The cicatricial lesions have mild consequences in the mouth, genitals and anus, but cause severe impairment in the esophagus, larynx, trachea, bronchi, and conjunctiva, which dictate more aggressive treatment and multidisciplinary management.

Esophageal stenosis, usually as a web located at the upper esophagus, causes the most severe damage to the esophagus. Esophageal stenosis causes dysphagia, weight loss and, at worst, malnutrition and/or false routes and pulmonary infection (34, 36–50).

Severe lesions at the nose and throat are perforation of the nasal septum and/or stenosis of nostrils, choanal, pharynx, and larynx (39, 51–53). Involvement of the trachea and bronchi may also rarely occur (51, 54). Scarring of the larynx or trachea are potentially life-threatening because this may lead to asphyxiation if tracheostomy is not performed. In general, mucous membrane lesions in EBA patients are, however, asymptomatic in 30% of cases (51).

Few case reports and small series of ocular involvement in EBA have been reported (13, 33, 34, 55–64). Patients presented the involvement of at least two other sites. Interestingly, in MM-EBA, immune deposits of IgA are present in half of the cases and the only Ig class in a third. Patients displayed a fibrosing conjunctivitis that might worsen and eventually cause blindness.

The frequency of esophageal, nasal and throat, and conjunctival involvement was, respectively, 6, 11, and 25% of 39 EBA cases in a French series (65). Bladder involvement has also been reported in one case (66).

In addition to MM-EBA, the involvement of mucous membranes in “skin-predominant” EBA is common (34). Hence, after the diagnosis of EBA, an interdisciplinary approach is needed for both diagnosis and treatment.

## IgA-EBA

Currently, IgA-EBA is defined as an EBA that presents with linear IgA deposits at the BMZ. IgA-EBA may resemble linear IgA bullous disease with erythematous cutaneous arciform lesions and a few scars and milium cysts, in particular in children. However, it IgA-EBA may also develop into a more severe clinical manifestation, especially with scarring at mucosal sites, as reported in 30% of the 82 cases in the Vodegel literature review, including 4% with severe ocular involvement (16, 19, 67, 68).

## Brunsting-Perry Type EBA

Patients with Brunsting-Perry type EBA present only cutaneous lesions, without erythematous or urticarial plaques, which predominate in the head and neck and heal leaving very atrophic scars. Review of seven of the eight cases reported in the literature has recently been published by Asfour et al. (69–76).

## Non-Inflammatory vs. Inflammatory Forms of EBA

The definition of inflammatory and non-inflammatory forms of EBA varies with the authors in the literature (17–21): (i) for most of them but one, the non-inflammatory form of EBA overlays the classical/non-mechano-bullous form; (ii) Buijsrogge et al. included the Brunsting-Perry like type in the mechano-bullous phenotype (19); (iii) for Briggaman et al. (17) who were the first to describe the inflammatory form of EBA, and for Kim et al. (18), the inflammatory form of EBA is synonymous to the BP-like form; (iv) for Buijsrogge et al. (19); and Iranzo et al. (20), all the patients who have not a mechano-bullous phenotype form, have an inflammatory phenotype and (v), for Seta et al. (21), inflammatory lesions are characteristic of the BP-like form of EBA but can also be seen in some patients with MM-EBA or IgA EBA.

The authors of the consensus conference (16) agreed on the following: (i) BP-like EBA are usually inflammatory forms of EBA, (ii) MM-EBA may be inflammatory forms of EBA, (iii) IgA-EBA may be inflammatory forms of EBA, (iv) Brunsting-Perry-like EBA are usually non-inflammatory forms of EBA, and (v) Brunsting-Perry-like EBA are not classical/mechano-bullous forms of EBA.

## Quality of Life in EBA and Associated Disorders

In general, EBA has a significant impact on the quality of life, which is now to be measured by generalized scores and the “autoimmune bullous disease quality of life” (ABQOL) and “treatment-based autoimmune bullous disease quality of life” (TABQOL) scores created specifically for autoimmune bullous diseases (77, 78).

Many systemic diseases have been reported to be associated with EBA, such as amyloidosis, thyroiditis, multiple endocrinopathy syndrome, rheumatoid arthritis, pulmonary fibrosis, chronic lymphocytic leukemia, thymoma, and diabetes [review in Gupta et al. (22)]. Most of these reports are, however, anecdotal. The only unarguable association of EBA with other diseases is with chronic inflammatory bowel diseases, in particular Crohn's disease, which has been reported to be present in 25% of EBA patients (65, 79). In B-cell lymphomas, presence of circulating and tissue-bound auto-antibodies to type VII collagen (COL7) has also been described in association with a frequency of 6% in 100 EBA cases, but the patients did not have clinical features suggestive of EBA (80). Furthermore, EBA associated with systemic lupus erythematosus, but not fulfilling the criteria of bullous erythematosus systemic lupus, are described (81, 82).

## Diagnosis

If clinically suspected, the minimal diagnostic criteria for EBA diagnosis are the detection of linear immunoglobulin- or C3-deposits along the dermal-epidermal junction in a perilesional skin biopsy with detection of a u-serrated pattern of Ig-binding.

**Figure 3:**
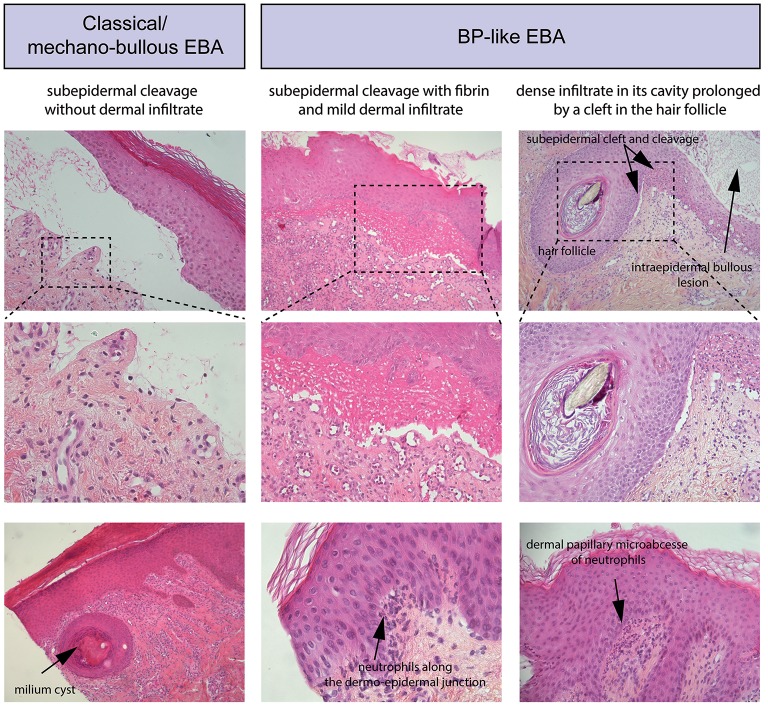
Standard histology. **(Left)** Histological study of lesional skin biopsies in a patient with a classical/mechanobullous form of EBA, subepidermal cleavage without dermal infiltrate; bottom, milium cyst in the dermis. **(Middle)** Patient with a BP-like form of EBA, subepidermal cleavage with fibrin in the blister cavity and mild dermal infiltrate. Bottom, Neutrophils along the dermo-epidermal junction **(Right)** Patient with a BP-like form of EBA, one intraepidermal bullous lesion and one subepidermal bullous lesion with dense infiltrate in its cavity prolonged by a cleft in the hair follicle; bottom, a dermal papillary microabcesse of neutrophils.

Routine histopathology from a lesional skin (or mucous membrane) biopsy does not allow to distinguish EBA from other subepidermal AIBD. It shows: (i) initially, papillary oedema and vacuolar alteration along the dermo-epidermal junction and at a later stage, a subepidermal or subepithelial cleavage, (ii) a great variability in the magnitude and/or quality of the inflammatory infiltrate, (iii) milia cysts and fibrosis in older lesions (Figure 3).

Definite diagnosis can be performed by either of the following methods: (i) serration pattern analysis of linear immunoglobulin deposits in the perilesional skin biopsy, (ii) fluorescent overlay antigen mapping (FOAM), (iii) immunoelectron microscopy, and/or (iv) detection of circulating antibodies against COL7.

If possible and needed, more than one of the above may be used to diagnose or exclude EBA. In addition, serology, i.e., detection of circulating anti-COL7 antibodies, should be performed, and, if positive, can serve as a biomarker of disease severity (83).

Diagnosis of EBA can be made by indirect immunofluorescence microscopy (IIF) using 1 M NaCl-split skin (SSS) as a substrate (84). Here, binding of antibodies to the dermal site (floor) of the blister is observed. By immunoblot analysis, binding to the 290-kDa antigen by the patient IgG is detected. The newly developed COL7 ELISA has a sensitivity of 45%. Combining SSS and ELISA reaches a sensitivity of 50%. Thus, half of the patients with EBA are sero-negative (85), and thus a negative serological finding does not exclude EBA as a differential diagnosis.

In serological negative cases, direct immunofluorescence (DIF) on sodium chloride-separated skin biopsy might reveal the diagnosis. Specifically, the diagnosis can be made using DIF serration pattern analysis, which shows distinct, EBA-specific, linear u-serrated immune-depositions at the BMZ (86). DIF serration pattern analysis by n-vs.-u may consider require expertise, which can be studied online: “n-vs.-u UMCG” (https://www.umcg.nl/NL/UMCG/Afdelingen/dermatologie/Wetenschappelijk_Onderzoek/NversusU/Paginas/default.aspx.

### Direct Immunodetection of Perilesional Skin Biopsies

**Figure 4:**
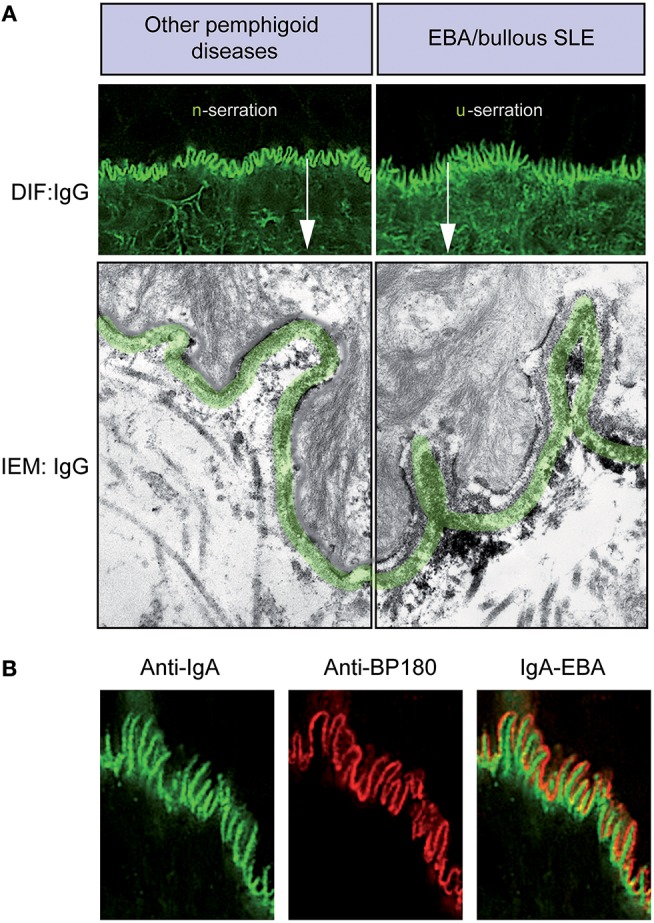
Diagnosis of EBA by direct immunofluorescence. **(A)** All pemphigoid variants are characterized by linear deposition of immunoglobulins and/or complement along the epidermal basement membrane zone. Serration pattern can be separated in an n-serrated pattern (blistering diseases with binding above the lamina densa with antibodies against hemidesmosomal components, e.g., BP, pemphigoid gestationis, mucous membrane pemphigoid, anti-p200 pemphigoid, and anti-laminin 332 pemphigoid, exept MM-EBA) and a u-serrated pattern (sublamina densa binding diseases caused by autoantibodies against COL7, e.g., EBA and bullous SLE). **(B)** In some cases, it is not possible to determine the serration pattern, especially in mucosal biopsies. In these cases, the level of the deposition of antibodies can be determined by fluorescent overlay antigen mapping (FOAM). FOAM is a technique that is based on the possibility of visualizing a targeted antigen relative to a topographic marker. For instance, using red staining, it is used for BP180 as a topographic reference marker, and green staining is used for IgA deposits. In the case of IgA-EBA, separate patterns of IgA deposits (green) and BP180 (red) can be observed with red staining on the dermal side.

All pemphigoid diseases are characterized by a linear deposition of immunoglobulins and/or complement along the epidermal basement membrane zone (Figure 4A). These antibodies are directed against various hemidesmosomal proteins: (i) type XVII collagen (BP180) in BP, MMP, pemphigoid gestationis, lichen planus pemphigoides, and LAD, (ii) BP230 in BP, (iii) laminin-332 in anti-laminin-332 pemphigoid, (iv) integrin β4 in ocular MMP, and (v) p200 in anti-p200 pemphigoid. Moreover, in EBA and bullous systemic lupus erythematosus (SLE), antibodies against COL7, present in the sublamina densa, also give rise to a linear deposition pattern (87).

If in suspected pemphigoid disease, a linear Ig- and/or C3- deposition is observed, it is important to determine the targeted autoantigen. In most variants of BP and in EBA, the deposits consist of IgG and complement. Mixed IgG/IgA depositions are usually encountered, especially in mucosal dominant pemphigoid. In some patients, IgA is the only present Ig-subtype, leading to a diagnosis of LAD or IgA EBA (67, 88, 89). However, in mucosal dominant pemphigoid with mixed IgA/IgG depositions, the IgG component may be faint, which sometimes result in a misdiagnosis of LAD. In very few patients, linear IgM deposition may be present in addition to IgG and C3. Even less cases have been described with linear IgM deposition only (90, 91).

Bullous SLE is characterized by antibodies against COL7 in patients fulfilling the diagnostic criteria for SLE. In bullous SLE, in addition to, or superimposed on a linear IgG deposition, a biopsy might show a lupus band, which is characterized by granular deposition of immunoglobulins and complement, in coincidence with epidermal anti-nuclear antibodies.

In most pemphigoid patients, a linear-serrated pattern can be discerned in direct IF microscopy of a peri-lesional skin biopsy. This serration pattern can be divided into an n-serrated and a u-serrated pattern (Figure 4A). The identification of these particular patterns allows to differentiate between (i) sublamina densa binding diseases caused by autoantibodies against COL7, e.g., EBA and bullous SLE and (ii) blistering diseases with binding above the lamina densa with antibodies against hemidesmosomal components, e.g., BP, pemphigoid gestationis, MMP, anti-p200 pemphigoid, and anti-laminin 332 pemphigoid. The u- and n-serrated patterns form based on the molecular architecture of the dermal-epidermal junction (DEJ): Specifically, if autoantibodies against COL7 are present, the immunodeposits are located between the rootlets of the basal keratinocytes, leading to the EBA-associated u-serrated pattern (Figure 4A). If, however, immune-depositions are located above the lamina densa, they trail the plasma membrane in the basal cell rootlets, resulting in the n-serrated pattern (Figure 4A). If it is not possible to determine the serration pattern, which occurs in few cases, it is recommended wise to cut thinner sections or obtain an additional peri-lesional skin biopsy.

However, if diagnosis of EBA cannot be established based on the serration pattern, the level of antibody deposition can be determined by fluorescent overlay antigen mapping (FOAM), which is based on the visualization of a targeted antigen relative to a known topographic marker. For instance, in Figure 4B, red staining is used for BP180 as a topographic reference, whereas green staining corresponds to IgA deposits. In the case of IgA-EBA, separate patterns of IgA deposits (green) and BP180 (red) can be seen with red staining on the dermal side (89). FOAM can be performed using a standard immunofluorescence microscope. However, confocal microscopy usually yields better results.

Direct immunoelectron microscopy provides a more detailed location of the deposition site, i.e., the lamina densa and/or sublamina densa at the DEJ (29). This location is distinct from that in other AIBDs (29, 30, 92).

### Indirect Immunofluorescence on Salt-Split Skin

Diagnosis of EBA can be made by indirect immunofluorescence (IIF) using 1 M NaCl-split skin (SSS) as a substrate. Here, antibody binding of to the dermal site (floor) of the blister is observed. By immunoblot analysis binding to the 290-kDa antigen of patient immunoglobulin is detected (84). Using SSS as a substrate, IIF shows IgG binding on the dermal side of the split. IgG binding can therefore be easily distinguished from BP and, in some cases, MMP, in which the immunoglobulins bind on the epidermal side of the split.

## COL7-Specific Serological Analyses by ELISA

For the serological diagnosis of EBA, three different assays are available: (i) an enzyme-linked immunosorbent assay (ELISA) that uses the non-collagenous (NC)1 and NC2 domains of COL7 (15, 93–95), (ii) an ELISA that is based on the NC1 domain alone (83), and (iii) an indirect IF test employing the NC1 domain (83). If they can be detected, serum levels anti-COL7 IgG correlate with disease activity (96). However, no correlation has been detected between the antibody specificity and the clinical phenotype (97).

## Therapy

First, the treatment of EBA remains challenging because no randomized control trials have been documented due to its rare prevalence (98, 99). Similar to other AIBDs, systemic corticosteroids are widely accepted as a first choice in the treatment of EBA. Initial doses range from 0.5 to 2.0 mg/kg/day (87). With systemic corticosteroids, steroid-sparing agents, including colchicine, diaminodiphenyl sulfone (DDS, dapsone), methotrexate (MTX), azathioprine (AZA), cyclosporine (CSA), mycophenolate mofetil (MMF), and cyclophosphamide (CPA), have been reported in treatment of EBA. Other therapeutic options, including high-dose intravenous Immunoglobulin (IVIG), rituximab (RTX), plasmapheresis and immunoadsorption (IA), and extracorporeal photochemotherapy (ECP) also have been reported (98, 100).

A retrospective analysis included 30 cases from the EBA cohort study regarding treatment and outcome (18). In this cohort study, the median time to remission was 9 months, and complete remission (CR) was observed in 33, 33, and 45% at 1 year, 3 years, and 6 years follow-up, respectively. Relapse sometimes occurs while receiving therapy, while a rate of relapse has not been reported in cohort studies.

Very recently, we collected information that included the treatment of EBA cases who met current diagnostic criteria published between 1971 and 2016 (101). Among all the reported treatments, we found IVIG and RTX to be associated with CR. Based on this retrospective analysis and previous reports in other autoimmune bullous diseases as a reference, each treatment is shortly summarized below.

## Colchicine

In EBA, colchicine is usually used at 1–2 mg/day. Of note, colchicine monotherapy (2 mg/day) has been reported (20, 102). By some experts, colchicine is considered a first line EBA treatment, especially for mild cases due to the relatively minor side effects compared with other therapeutic choices (103–105). There is currently no controlled study reporting the efficacy of this treatment, and no experimental data are available. Hence, colchicine is a triable treatment for mild cases, and it should be considered if other immunosuppressant treatments are ineffective.

## Diaminodiphenyl Sulfone (DDS, Known as Dapsone)

Based on expert opinion, DDS is considered a safe and relatively effective first line treatment (18). Although DDS monotherapy (100 mg/day) has been reported with CR in single IgA-EBA cases (67), 25–150 mg/day of DDS is usually used as an adjuvant therapy with systemic corticosteroids. Adverse effects, haemolysis, methemoglobinemia, agranulocytosis, peripheral neuropathy, and psychosis might be observed (106). A rare adverse effect called “dapsone syndrome” should be considered. Some experts recommend that this therapy can be effective (105, 107). Similar to BP (108), DDS appears to have a corticosteroid-sparing effect in EBA.

## Methotrexate (MTX)

Usually, MTX is used in combination with systemic corticosteroid with/without other immunosuppressants at 20–25 mg weekly. A retrospective review for MTX in the treatment of pemphigus and pemphigoid has reported the use of 5–50 mg weekly of MTX. Adverse effects, including nausea, anemia, and infection sometimes led to the discontinuation of treatment (109). In EBA, no case-series study has focused on the efficacy of MTX. Based on the efficacy in BP (109), MTX may be a viable treatment for EBA as a corticosteroid-sparing agent.

## Azathioprine (AZA)

In EBA, AZA was the most frequently used immunosuppressant as an adjuvant therapy (101). Two types of adverse effects have been reported; non-dose-related adverse effects, including pancreatitis, fever, rash, malaise, nausea, diarrhea, and hepatitis, and dose-related adverse effects, including leucopoenia and some forms of hepatitis (110). Like MTX, no case-series study has focused on the efficacy of AZA in EBA. Based on the efficacy in BP (108), AZA may not be a beneficial treatment for EBA, and further analysis is needed.

## Cyclosporine (CSA)

CSA is mainly used as an adjuvant therapy (101). Renal dysfunction, hypertension, headache, tremor, paraesthesia, hypertrichosis, and hyperlipidaemia may be observed as adverse effects, and most persistent renal dysfunction is related to prolonged therapy or doses >5 mg/kg/day (111).

## Mycophenolate Mofetil (MMF)

In our recent, retrospective analysis (101), 1–3 g/day of MMF was used in combination with systemic corticosteroids. A randomized clinical trial of methylprednisolone plus-AZA vs. -MMF therapy in pemphigus showed a slightly lower frequency of adverse effects, including hypertension, hyperglycaemia, and infection in the MMF-treated group, although it was not a significant difference (112). The results from the randomized control study in pemphigus suggested that 2 g/day offered a better risk–benefit profile than 3 g/day (113). In a case-series study, EBA cases were successfully treated with MMF as a steroid-sparing agent (114). Hence, MMF could be a steroid-sparing agent in EBA.

## Cyclophosphamide (CPA)

There has been no case-series study focused on the efficacy of CPA in EBA, and relativity fewer reports have examined treatment with CPA compared with other immunosuppressants. Therefore, CPA appears to be a therapeutic option when other immunosuppressants cannot control the disease, but limited data is available (101).

## High-dose Intravenous Immunoglobulin (IVIG)

In our recent, retrospective analysis (101), 31 EBA cases were treated with IVIG, among which 24 cases providing information on the regimen of IVIG and outcome are summarized in Table 2. Ahmed et al. reported 10 cases treated with 2 g/kg/cycle (divided into 3 consecutive days) of IVIG in EBA with severe disease and non-responsive to conventional therapies (118). After 16–22 cycles of IVIG therapy, clinical remission was observed from 29 to 123 (mean 53.9) months without any other therapies. The main adverse effect was headache, which increased the intervals of each infusion in two cases, although no severe adverse effects were observed. These two cases are also well summarized in previous reports of IVIG treatment in EBA. In most cases, 2 g/kg/cycle for 3 days or 400 mg/kg/day for 5 sequential days were used with clinical improvement. In experimental mouse studies of EBA and BP, beneficial effects of IVIG have been demonstrated (128–131). Recently, a randomized double-blind trial of IVIG was reported in BP showing a lower disease activity score in the IVIG-treated group compared with placebo (132). Therefore, IVIG is an effective treatment in severe or intractable cases of EBA.

**Table 2:** Reports of IVIG treatment in EBA.

**Reported year**	**Reference**	**Age**	**Sex**	**Phenotype of EBA**	**Treatments prior to IVIG**	**Regimen of IVIG**	**Concomitant-started treatment**	**Outcome**
2013	(115)	37	f	BP-like	Corticosteroid, colchicine, DDS	2 g/kg/cycle		No response
2013	(116)	20	f	BP-like	Corticosteroid, DDS	500 mg/kg/day, 4 days	colchicine (1 mg/day)	CR
2013	(117)	2	m	BP-like	Corticosteroid	400 mg/kg/day, 4 days	DDS (1 mg/kg/day)	PR
2011	(118)	55	f	BP-like	Corticosteroid, AZA	2 g/kg/cycle divided in 3 days		CR
2011	(118)	61	m	Mechanobullous	Corticosteroid, DDS	2 g/kg/cycle divided in 3 days		CR
2011	(118)	37	m	Mechanobullous	Corticosteroid, DDS	2 g/kg/cycle divided in 3 days		CR
2011	(118)	55	f	Mechanobullous	Corticosteroid, DDS	2 g/kg/cycle divided in 3 days		CR
2011	(118)	47	f	Mechanobullous	Corticosteroid	2 g/kg/cycle divided in 3 days		CR
2011	(118)	50	f	Mechanobullous	DDS, MMF, MTX	2 g/kg/cycle divided in 3 days		CR
2011	(118)	73	m	Mechanobullous	Corticosteroid, DDS	2 g/kg/cycle divided in 3 days		CR
2011	(118)	75	f	Mechanobullous	Corticosteroid, DDS	2 g/kg/cycle divided in 3 days		CR
2011	(118)	59	m	BP-like	Colchicine, CSA	2 g/kg/cycle divided in 3 days		CR
2011	(118)	62	f	BP-like	Colchicine, MTX, MFM	2 g/kg/cycle divided in 3 days		CR
2007	(119)	70	m	Unknown	Corticosteroid, AZA, DDS, CSA	2 g/kg/cycles		PR
2007	(120)	65	m	Mechanobullous (+p200 pemphigoid)	Corticosteroid, DDS, CSA, MMF	400 mg/kg/day, 5 days		PR
2007	(121)	58	f	Unknown	Corticosteroid, AZA, MMF, CSA	2 g/kg/cycles		no response
2006	(63)	22	m	Unknown	Corticosteroid, DDS	2 g/kg/cycle divided in 3 days		PR (4 cycles later)
2006	(122)	54	f	Mechanobullous	Corticosteroid, AZA, colchicine,	2 g/kg/cycle divided in 5 days		PR (4 cycles later)
2002	(123)	43	f	Both	None	400 mg/kg/day, 5 days		CR (PR after 1 cycle)
2000	(124)	37	m	BP-like	Corticosteroid	1.2 g/kg/cycle divided in 2–3 days		CR (after 9 months)
1998	(46)	59	m	Mechanobullous	Corticosteroid, DDS, MTX, CSA, CPA, IA	400 mg/kg/day, 5 days		CR
1997	(125)	29	m	BP-like	Corticosteroid, AZA, DDS, PE, colchicine, CSA	40 mg/kg/day, 5 days		CR (after 4 cycles)
1995	(126)	55	m	BP-like	Corticosteroid, AZA, DDS, colchicine	400 mg/kg/day, 5 days		CR (after 9 cycles)
1993	(127)	16	m	Both	Corticosteroid, CSA	400 mg/kg/day, 4 days every 2 weeks		CR

*BP, bullous pemphigoid; DDS, diaminodiphenyl sulfone; MTX, methotrexate; AZA, azathioprine; CSA, cyclosporine; MMF, mycophenolate mofetil; CPA, cyclophosphamide; IVIG, high-dose intravenous immunoglobulin; PE, plasma exchange; CR, complete remission; PR, partial remission*.

## Rituximab (RTX)

Rituximab, a humanized anti-CD20 monoclonal antibody, was used in 10 cases with a protocol of 375 mg/m^2^ weekly for 4 weeks (in most cases) and was associated with CR in our recent, retrospective analysis (101), which is summarized in Table 3. In pemphigus, another regimen, 1,000 mg every 2 weeks on day 0 and day 14 twice and 500 mg at 12 and 18 months, showed good efficacy in an open-label randomized trial (140). A recent study reported 4 EBA patients treated with 1,000 mg of RTX every 2 weeks twice, similar to the regimen in pemphigus with PR and CR outcomes in each case and no response in 2 cases (141). In EBA animal models, depletion of B cells at the induction of experimental disease showed that B cells, in addition to developing into plasma cells, serve as important antigen-presenting cells. Specifically, if anti-CD20 treatment was applied at the time of immunization, development of antigen-specific CD4+ T cells was significantly hampered in immunization-induced EBA (142). Hence, RTX seems to be a promising treatment option for EBA. Further controlled clinical studies are required to determine which regimen of RTX is most effective in EBA.

**Table 3:** Reports of RTX in EBA.

**Reported year**	**Reference**	**Age**	**Sex**	**Phenotype of EBA**	**Treatments prior to RTX**	**Regimen of IRTX**	**Concomitant-started treatment**	**Outcome**
2013	(133)	71	f	Unknown	corticosteroid, DDS	375 mg/m^2^, every week, 4 w, 4 cycles	IA	CR (after 18 weeks)
2012	(134)	68	f	Mechanobullous	Corticosteroid, colchicine, MTX, DDS, AZA	375 mg/m^2^, every week, 4 w		CR (after 16 weeks)
2010	(43)	50	m	Mechanobullous	Corticosteroid, MMF, colchicine, IVIG, AZA	375 mg/m^2^, every week, 4 w		CR (over 4 months)
2010	(135)	71	f	BP-like	Corticosteroid, DDS, colchicine	375 mg/m^2^ every week, 4 w, 1 cycle	IA	CR (within 16 weeks)
2009	(136)	54	f	Mechanobullous	Corticosteroid, dapsone, aza, CSA, CPA, IVIG, MMF	375 mg/m^2^, every week, 4 w, 3 cycles		PR (CR for skin involvements)
2007	(137)	75	f	BP-like	Corticosteroid, AZA, MMF	375 mg/m^2^, every week, 4 w		PR (for 10 months)
2007	(138)	67	m	Mechanobullous	Corticosteroid, AZA, CSA, DDS, MTX, CPA, ECP	375 mg/m^2^, every week, 4 w	IA	PR
2007	(138)	42	m	Mechanobullous	Corticosteroid, AZA, CSA, DDS, MTX, CPA, IVIG	375 mg/m^2^, every week, 4 w	IA	PR
2007	(121)	58	f	Mechanobullous	Corticosteroid, AZA, MMF, CSA, IVIG	375 mg/m^2^, every week, 4 w		PR (after 1 week)
2006	(139)	46	m	BP-like	Corticosteroid, DDS, AZA, IA, colchicine	375 mg/m^2^, every week, 4 w		CR (after 11 weeks)

*DDS, diaminodiphenyl sulfone; MTX, methotrexate; AZA, azathioprine; CSA, cyclosporine; MMF, mycophenolate mofetil; CPA, cyclophosphamide; IVIG, high-dose intravenous immunoglobulin; RTX, rituximab; PE, plasma exchange; IA, immunoadsorption; ECP, extracorporeal photochemotherapy, CR, complete remission; PR, partial remission*.

## Plasmapheresis and Immunoadsorption (IA)

Although plasmapheresis has been used for the treatment of pemphigus and pemphigoid, including EBA, it makes the shift to IA because of its advantages compared with plasmapheresis: (i) selective removal of immunoglobulin from the circulation; (ii) no requirement for the substitution of plasma components, such as human albumin or fresh frozen plasma; (iii) two to three times more processing capacity per treatment session than plasmapheresis; and (iv) fewer side-effects, such as infections and allergic reactions (90). Interestingly, there are several reports of combination therapy with IA and RTX in EBA that might provide an effective treatment protocol in EBA (133, 135, 138). Kolesnik et al. (133) and Kubisch et al. (135) reported that each patient was treated with IA for 3 consecutive days followed by IA every week and 375 mg/m^2^ of RTX on the day after IA for 4 weeks, leading CR after 18 months and CR within 16 weeks, respectively. Niedermeier et al. (138) reported 2 intractable cases treated with 2 cycles of IA for 4 consecutive days at 4-week intervals followed by RTX (375 mg/m^2^, every week for 4 w), leading to PR. Interestingly, antigen-specific immunoadsorption, i.e., where only autoantibodies specific for the respective autoantigen are removed from the circulation, are in pre-clinical development (143, 144).

## Extracorporeal Photochemotherapy (ECP)

ECP has been reported in the treatment of Sezary syndrome, mycosis fungoides, and autoimmune bullous diseases (145). There are several reports of the use of ECP in refractory EBA with outcomes of CR and PR in 3 cases, respectively, and no response in 1 case (61, 100, 146, 147). The mode of action of ECP in the treatment of EBA is still unknown, although one report has shown a decrease in circulating antibody detected by immunofluorescence and an increase in suction blister time (100). Despite the low number of published EBA patients, due to the reported success rates, ECP should be considered a therapeutic option in patients with refractory EBA.

## Others

Daclizumab, a humanized monoclonal antibody against the a-subunit of the high-affinity interleukin-2 receptor also known as the Tac antigen or CD25, was reported in the treatment of EBA (148), in which only one of 3 cases showed clinical improvement. Sulfasalazine was used in a patient with EBA associated with Crohn's disease, resulting in no improvement of the skin lesion (149). The usefulness of doxycycline has been reported in BP (150). Doxycycline and another tetracycline, minocycline, were found in the literature on EBA cases, although its usefulness remains unclear in EBA treatment (151–153).

## Prognosis

EBA is a chronic disease characterized by exacerbations and remissions over the course of months to years. Although data on the prognostic factors in EBA are lacking in the literature, the experts admit that the prognosis of EBA depends on its severity at the time of diagnosis and propose treatment accordingly.

Analogous to BP (154, 155) and MMP (35), an EBA is considered severe if the patient has 10 or more cutaneous bullous lesions and/or 3 or more instances of mucosal sites and/or conjunctival, laryngo-tracheal or esophageal involvement. Otherwise, the EBA is classified as moderate or minimal. The MMP-DAI (disease activity index) score (156) can be used to quantify the extent of the disease, but the cut-offs between the severe, moderate, and minimal forms of EBA have not been established to date.

The goal of the treatment is to obtain control of the disease followed by CR, i.e., the absence of active lesions (erythema, urticaria, bullous lesions, and erosions) without worsening of the cicatricial lesions, which are irreversible.

A CR off treatment of EBA is not possible since a long-term maintenance treatment is recommended. CR under minimal treatment may occur after months to years in mild or moderate forms (unpublished data), but minimal skin fragility without bullous lesions can persist for several months to years. The milium cysts may eventually disappear.

The prognosis has been reserved in severe forms, as evidenced by the publication of numerous case reports in therapeutic failure. Indeed, in some patients, the disease may progress quickly with periods of severe exacerbation and rapid scarring. The cicatricial lesions (synechiae, stenosis, joint contractures) may engage the functional prognosis and be life-threatening. In a retrospective study of 30 patients with EBA, all of whom were initially treated with a combination of methylprednisolone, dapsone, and colchicine (six who did not respond were subsequently treated with other immunosuppressants), 8 of 24 patients (33 percent) achieved complete remission and 5 of 24 (21 percent) achieved partial remission within 1 year (18). The prognosis of these severe forms could improve because of recent publications demonstrating the therapeutic success of intravenous immunoglobulins and rituximab (see above). The overall prognosis and response to treatment may be more favorable in children than in adults (157, 158).

Taken together, these findings underline the need for an early diagnosis, multidisciplinary care by experienced practitioner and prompt implementation of appropriate treatment to improve the prognosis of EBA.

## Pathogenesis

### COL7 as the Autoantigen in EBA

Nearly a century after the first description of EBA, the carboxyl terminus of COL7 was identified as the autoantigen in EBA. Since that time, it has been shown that most patients develop autoantibodies that bind to epitopes located within the NC1 domain of COL7 (159–162), whereas antibody reactivity to either the collagenous domain (163) or the NC2 domain (164) is detected in a very small minority of patients. No correlation was detected between antibody specificity and clinical phenotype (159). In a recent multicentre study with 95 EBA patients, NC1/NC2 ELISA showed a higher sensitivity (97.9%) than NC1 ELISA (89.5%), supporting a considerable number of patients with antibodies against NC2 (95).

Interestingly, the humoral autoimmune response toward COL7 encompasses almost all IgG subclasses. Most commonly, COL7 autoantibodies are IgG, but in ~10% of EBA patients, IgA autoantibodies against COL7 are detected. Few cases of IgE- and IgM-COL7-reactive immunoglobulins have been described (101). The nature and/or cause of this broad immunoglobulin isotype reactivity against COL7 is, however, unknown.

## Genetic and Environmental Factors Contribute to Tolerance Loss in EBA

As with most autoimmune diseases, the exact cause of the disease is unknown. With regard to EBA, the data indicate a certain genetic predisposition as well as a contribution of environmental factors to EBA pathogenesis. Due to the small number of EBA cases, it is difficult to study the influence of certain environmental factors or infections. EBA susceptibility is associated with genes in and outside the major histocompatibility complex (MHC) locus. Specifically, an association with the MHC locus (HLA-DR2) has been documented in humans in two independent studies (10, 11). The association with the MHC locus is also supported by animal studies, where an association of susceptibility to immunization-induced EBA is linked to the H2s locus (165). Evidence for the involvement of genes outside the MHC locus arises from one case of coincident EBA in members of a family provided further support for the genetic control of EBA (166). The contribution of genes outside the MHC locus is again underscored by corresponding observations in experimental EBA (165, 167, 168). First, when C57Bl6/J mice are immunized with COL7, they develop autoantibodies but no clinical disease. When mice on the same genetic background lack expression of the inhibitory Fc gamma receptor (FcγR) IIB, they also develop clinically overt blistering (165, 168). Similarly, mice carrying the EBA-associated H2s allele develop severe clinical disease when on the B6 genetic background but only moderate disease when on the C57BL/10 background (165, 169). To pinpoint the mutations associated with EBA susceptibility, mice of an advanced, autoimmune-prone intercross line were immunized with COL7. Herein, one third of the mice developed clinical disease, while the remaining mice remained phenotypically healthy (170, 171) Classical quantitative trait loci mapping identified several genes outside the MHC that were associated with either the onset or severity of clinical disease (170). However, the number of genes is still too large to pinpoint the association with clinical disease to single genes, yet in a nutshell, it provides evidence for a genetic basis of EBA susceptibility.

**Figure 5:**
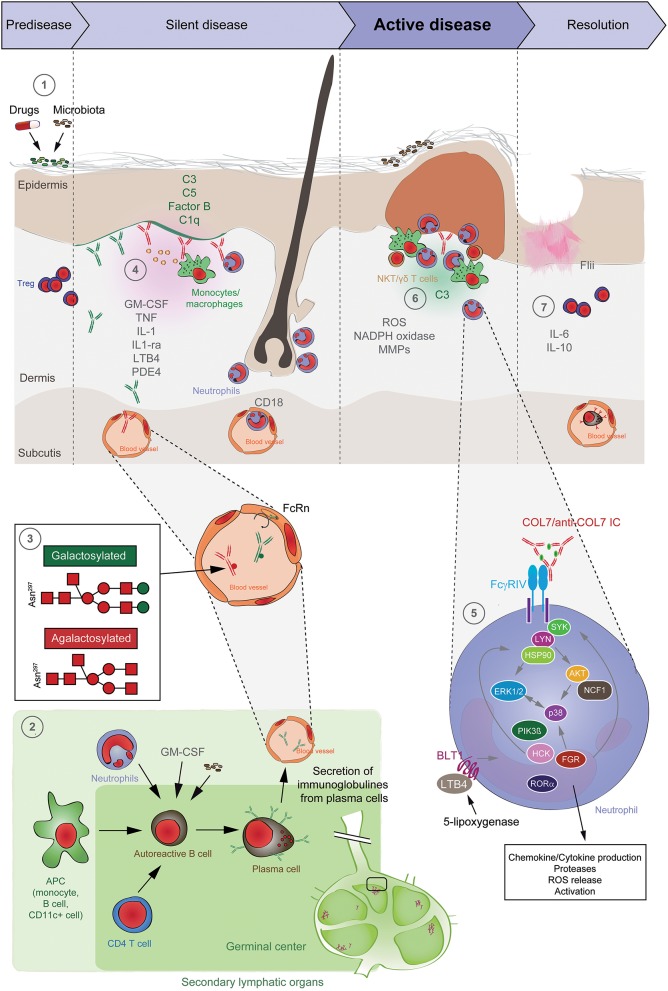
Pathogenesis of EBA. (1) Genetic factors and the skin microbiome promote a tolerance loss. (2) This phenomenon is mediated by the interaction of APCs with autoreactive B and T cells, leading to clonal expansion and differentiation into plasma cells. Autoantibodies against COL7 are released into the blood circulation and effector organs. (3) During inflammation, galactosylation of antibodies may differ. High galactosylation of IgG is crucial for these anti-inflammatory properties, whereas low galactosylation is pro-inflammatory. (4) Binding of autoantibodies to DEJ in the skin induces complement deposition, pro-inflammatory cytokine and mediator release and subsequently leukocyte extravasation. (5) Immune complexes bind in a Fc-dependent manner to neutrophils and induce a signaling cascade leading to activation, including the (6) release of ROS and matrix metalloproteases. In addition to neutrophils, other cell types are involved in split formation, as shown for monocytes/macrophages, NKT and γδ T cells. By contrast, T_reg_ cells have an inhibitory effect on EBA progression. (7) Resolution of autoantibody-induced tissue injury. T_reg_, regulatory T cell; NKT, natural killer cell; C, complement; GM-CSF, granulocyte-macrophage colony-stimulating factor; IL, interleukin; LTB4, leukotriene B4; PDE4, phosphodiesterase 4; ROS, reactive oxygen species; NADPH, nicotinamide adenine dinucleotide phosphate; MMPs, matrix metalloproteinases; APC, antigen-presenting cell; CD, cluster of differentiation; SYK, spleen tyrosine kinase; Lyn, tyrosine-Protein Kinase Lyn; HSP, heat shock protein; AKT, protein kinase B; NCF1, neutrophil cytosolic factor 1; ERK, extracellular signal-regulated kinase; PI3K, phosphatidylinositol-4,5-bisphosphate 3-kinase; HCK, tyrosine-protein kinase HCK; FGR, tyrosine-protein kinase FGR; RORα, retinoid-related orphan receptor-alpha; BLT1, leukotriene B4 receptor 1; LTB4, leukotriene B4.

In addition to genetic factors, animal models of EBA clearly indicated an influence of resident microbial communities in disease pathogenesis (172, 173) (Figure 5). By the use of outbred mice in immunization-induced EBA, it could be shown that Firmicutes were the most abundant (54%), followed by Proteobacteria (21%), Actinobacteria (12%), and Bacteroidetes (6%), which is similar to previous studies in the skin. At the genus level, Staphylococcus (36%), Corynebacterium (9%), and Ralstonia (8%) were most abundant (172). Ellebrecht et al. used the same model to show skin community changes before and after immunization. Among SJL/J mice that were immunized with COL7, only 80% of the mice developed disease, whereas the others remained healthy. Interestingly, the specific antibody concentrations and binding of antibodies to the DEJ were unaffected. By contrast, immunized mice that did not develop clinical phenotypes showed a greater alpha diversity, compared to mice that developed EBA symptoms after immunization (173).

## The Afferent Phase: CD4 T Cell-Dependent Production of Auto-Antibodies Against COL7

EBA is characterized and causes autoantibodies directed against COL7. Unfortunately, little human data are available for this relevant and complex phase of pathogenesis, which includes the interaction of various immune cells, such as antigen-presenting cells, autoreactive B cells, T cells and neutrophils, and subsequently leads to antibody production. Regarding human data, COL7-specific T cells (isolated from the blood) can be detected EBA patients (174, 175), but most data concerning the interaction of these cell in the afferent phase has been derived from animal models (167).

In the immunization-induced EBA model, T-cell-deficient mice do not develop COL7-specific antibodies and, consequently, clinical disease development, indicating that autoantibody production in this model is T cell-dependent. Furthermore, disease susceptibility could be restored in T cell-deficient SJL/J mice by T cells transfer from COL7-immunized wild-type mice (176). To delineate T-cell subsets involved in the generation of anti-COL7 antibodies in this model of immunization-induced EBA, CD4+ and CD8+ T cells were depleted for 2 weeks, starting at immunization. Depletion of CD4+ T cells led to a delay of both autoantibody production and the clinical disease onset. By contrast, CD8+ T cells depletion at the same time period did not impact production of COL7-specific autoantibodies or clinical disease (142). Therefore, in experimental EBA, CD4+ T are required for induction of autoantibody production. Since only few and specific inbred mouse strains developed clinically overt disease after COL7 immunization, the autoantibody response of clinically healthy vs. diseased mice after immunization was contrasted. Here, complement-fixing antibodies were linked to clinical EBA manifestation (169). Furthermore, by determination of the IgG isotype of the autoantibodies, a Th1 polarization of the immune response was noted. In addition, an increase in the Interferon (IFN)-γ/Interleukin (IL)-4 ratio in the draining lymph nodes of EBA-susceptible mice compared with EBA-resistant strains was observed (169). Regarding the involvement of neutrophils in autoantibody production, GM-CSF-deficient mice generated less COL7-specific autoantibodies, which was paralleled by reduced neutrophil numbers in peripheral lymph nodes, draining the immunized site. The same effect was observed in neutrophil-depleted wild-type mice (177).

To further address which antigen-presenting cells are required the formation of antigen-specific CD4+ T cells, B cells were depleted in mice and subsequently immunized with COL7. In the absence of B cells, the antigen-specific CD4+ T-cell response was completely abolished. Furthermore, depletion of dendritic cells and macrophages had similar effects. Hence, the development of COL7-specific CD4+ T cells requires the presence of APC—specifically B cells, dendritic cells, and macrophages (142) (Figure 5). More interestingly, the absence of T_reg_ cells in scurfy mice cells led to blistering via the formation of pathogenic autoantibodies, demonstrating a critical involvement of these cell types in the afferent phase of EBA (178, 179).

In contrast to T cells, autoreactive B cells are almost exclusively found in the peripheral lymph nodes in the immunization-induced EBA mouse model (180, 181), which may be due to missing expression of homing-associated CXCR3 and CXCR4 chemokine receptors. In immunization-induced EBA, COL7-specific plasma cells have a half-life of ~7 weeks. This resembles an intermediate between short- and long-living plasma cells (180). A similar intermediate plasma cell type is most likely also present in patients because autoantibody titers in patients with autoimmune bullous diseases slowly decline over a period of 8–12 weeks after B cell-depleting rituximab treatment (182).

An important molecular requirement for autoantibody production in experimental EBA is heat-shock protein 90 (Hsp90). Specifically, pharmacological HSP90 inhibition suppressed autoantibody production in immunization-induced EBA. In the same model, HSP90 blockade impaired the onset of clinical disease manifestation when injected prior to immunization with COL7. Furthermore, clinical disease progression was ameliorated when the compounds were applied in therapeutic experimental settings. Interestingly, B cell development was unaffected by the HSP90 inhibition, while T-cell proliferation was impaired. Overall, this identified T cells as targets of HSP90 inhibition in experimental EBA (174).

## Circulation and Pathogenicity of Autoantibodies Against COL7

### Targeting the Half-Life of Anti-COL7 Autoantibodies

After (auto)-antibodies are present in the circulation, their half-life is controlled by the neonatal Fc receptor (FcRn). FcRn is constructed as a heterodimer, consisting of an alpha-chain and a beta-2-microglobulin light chain (183). Among other functions, FcRn protects IgG from catabolism (184). Inhibition of the FcRn leads to the enhanced clearance of IgG, including autoantibodies. In antibody transfer-induced animal models of EBA (167), disease induction in mice is completely blocked (128, 185). However, this protection can be overridden by the transfer of large amounts of antibodies (185). Similarly, treatment of experimental EBA or other pemphigoid diseases with high doses of IgG (IVIG), which by saturation also inhibits the FcRn, reduces circulating autoantibody titers and leads to disease improvement (128, 129, 186, 187), although FcRn inhibition is most likely not the only mode of action of IVIG in EBA (131, 188, 189).

Interestingly, two clinical trials are currently being conducted to evaluate the safety and efficacy of FcRn inhibitory treatments in patients with other autoimmune skin blistering diseases (NCT03334058) (190). Hence, this may become a treatment option for EBA patients in the not too distant future (167).

### Targeting the Pathogenicity of Anti-COL7 Autoantibodies

In addition to its half-life, the pathogenicity of an antibody can also affect disease progression. In general, IgG antibodies have one conserved N-glycosylation site in each of their constant heavy chain regions. These Fc glycans have a major impact on their structure as well as their effector functions. Non-galactosylated (agalactosylated; G0) IgG antibodies have long been thought to have pro-inflammatory effector functions in autoimmune patients with rheumatoid arthritis. In contrast, sialylated IgGs mediate anti-inflammatory effects. Recent evidence also suggests that pro-inflammatory immune responses, including autoimmune reactions, mainly induce antigen-specific G0 IgGs, whereas tolerance leads to the generation of immunosuppressive, galactosylated, and sialylated IgGs. Under normal conditions, differentially glycosylated IgGs clearly mediate their pro- or anti-inflammatory effector functions as immune complexes in an antigen-specific manner (191) (Figure 5). In agreement with these findings, the use of EndoS, an endoglycosidase derived from Streptococcus pyogenes that selectively hydrolyses the N-linked glycan of native IgG, impaired split formation at the DEJ in skin cryosections. In EBA mouse models, EndoS abrogated clinical disease induction in mice (192, 193). This observation raises the possibility that the glycosylation status of IgG can also affect the onset, severity and progression of disease.

## The Efferent Phase of EBA: Attraction and Activation of Leukocytes Leads to Blister Formation

Based on the current understanding of EBA pathogenesis (Figure 5), the effector phase of EBA, i.e., autoantibody-induced inflammation and blistering, can be divided into (**i**) autoantibody binding to COL7, (**ii**) complement activation and the formation of a pro-inflammatory milieu, (**iii**) leukocyte extravasation, (**iv**) activation by Fcγ receptors, and (**v**) tissue damage. Mechanisms leading to non-inflammatory blistering in EBA are, in contrast, poorly understood. With the increased understanding of pathomechanisms of epitope-dependent pathogenicity-associated (194), non-inflammatory BP, new insights into mechanobullous EBA can be expected. However, due to a lack of data on non-inflammatory mechanisms of blistering in EBA, the following text relates to inflammatory EBA.

Autoantibody-induced tissue injury in EBA is initiated by **(i) the deposition of autoantibodies at the DEJ**. Apart from the skin anti-COL7 antibodies bind to the esophagus, stomach, small intestine, and colon because of the autoantigen expression at these sites (195, 196). Yet, not all isotypes of anti-COL7 have the potential to induce dermal-epidermal separation: *Ex vivo*, only human IgG1 and IgG3, but not IgG2 and IgG4, are capable to cause blistering (197). Furthermore, immune complexes containing IgA1 or IgA2 COL7 autoantibodies activate neutrophils and also induce subepidermal blistering when in cryosections of human skin. Of note, and in contrast to IgG1 autoantibodies, neither IgA1 nor IgA2 leads to complement deposition at the dermal-epidermal junction. Because complement activation has traditionally been thought a prerequisite for blister induction, this may be compensated by so far unknown soluble factors and/or by a stronger activation of neutrophil granulocytes when engaging IgA immune complexes (198).

Thereafter, **(ii) a pro-inflammatory milieu is generated** in the skin, which includes activation of the complement system (199). The complement system consists of circulating proteins that, upon activation, initiate a highly controlled cascade that is an integral part of the innate humoral immune response (200). C5-deficient mice (168) are either completely or (168) partially (201) protected from induction of experimental EBA by antibody transfer.

Dissecting the specific role of each complement activation pathway (classical, lectin, and alternative pathway) showed the following: MBL deficient mice showed a similar EBA phenotype to the wild-type controls. C1q-deficient mice showed weak and partial protection, while factor-B-deficient mice showed clinically relevant protection from EBA induction by antibody transfer (199). This identified the alternative pathway of the complement system as a main driver of skin blistering and inflammation in antibody transfer-induced EBA (202). Downstream of C5, C5ar1-deficient mice are significantly protected from experimental EBA, whereas C6-deficient mice developed widespread blistering disease, excluding the membrane attack complex as a pharmacological target for EBA. In line, pharmacological blockade of C5, factor B, or C5aR1 led to a significant improvement of the blistering phenotype in antibody transfer-induced EBA (202, 203).

In addition to the complement system, the lipid mediator leukotriene B4 (LTB4) is a potent granulocyte chemoattractant (204, 205) and activator (206) and is abundant in the blister fluids of bullous pemphigoid patients, but its pathogenic significance for pemphigoid diseases had been unknown until recently. LTB4 is biosynthesized from arachidonic acid through sequential enzymatic conversion by 5-lipoxygenase and LTA4-hydrolase. The 5-lipoxygenase is most abundant in neutrophils, and it is activated upon cell stimulation by, for example, immune complexes or the complement fragment C5a (207). Mice deficient in 5-lipoxygenase, a key enzyme in LTB4 biosynthesis, or BLT1-deficient mice are completely resistant to the induction of experimental EBA by antibody transfer (208). In addition to complement and lipid mediators, several cytokines have been identified to modulate the effector phase of EBA. Cytokines that are differentially regulated in experimental EBA (209) or cytokines associated with those functional data (210) are summarized in Table 4 (171, 224).

**Table 4:** Mediators of the EBA effector phase.

**Target**	**Function**	**References**
C5	C5-deficient mice are partially or completely protected from EBA inducing by antibody transfer	(201, 211)
C1q/factor B	Respective knock-out mice are partially protected from EBA inducing by antibody transfer	(203)
IgG glycosylation	Enzymatic removal of terminal IgG N-glycosylation renders anti-COL7 antibodies non-pathogenic in antibody transfer-induced EBA	(192, 193)
Galactosylated IgG	Immune complexes with highly galactosylated immune complexes inhibit pro-inflammatory signaling of the C5aR1 through dectin-1 and Fc gamma receptor IIB, resulting in a protection from antibody transfer-induced EBA	(202)
IL-6	In antibody transfer-induced EBA, IL-6 has anti-inflammatory effects, through up-regulation of IL-1ra	(209)
CXCR-1/2	Blockade of the CXCR-1/2 ligands impairs induction of EBA by antibody transfer and slows disease progression when applied in therapeutic settings in immunization-induced EBA	(212)
GM-CSF	Blockade of GM-CSF impairs induction of EBA by antibody transfer and slows disease progression when applied in therapeutic settings in immunization-induced EBA	(177)
IL-1/IL-1ra	Both anti-IL1β or IL-1ra (anakinra) treatment impair the induction of EBA by antibody transfer. Additionally, anakinra halts disease progression when applied therapeutically in immunization-induced EBA	(213)
TNFa	Blockade of TNF impairs induction of EBA by antibody transfer and halts disease progression when applied in therapeutic settings in immunization-induced EBA	(214)
LTB4	Blockade of either LTB4 biosynthesis or its' receptor completely protects mice from EBA induction by antibody transfer	(208)
IL-17A/E	IL17R-deficient mice are partially protected from EBA inducing by antibody transfer	(215)
NADPH oxidase	Neutrophil cytosolic factor 1-deficient mice, lacking functional NADPH oxidase, -deficient mice are completely protected from EBA inducing by antibody transfer	(216)
Elastase	Elastase is required for the induction of subepidermal blisters *ex vivo*	(217)
Flii	Blockade of Flii protects mice from EBA induction by antibody transfer	(218–220)
MIP1α	Increased expression, but no effect on clinical phenotype	(221)
S100	Increased expression, but no effect on clinical phenotype	(222)
Trem1	Increased expression, but no effect on clinical phenotype	(223)

*C, complement factor; IgG, immunoglobulin G; IL, interleukin; COL7, type VII collagen; CXCR, CXC-chemokin receptor; GM-CSF, granulocyte-macrophage colony-stimulating factor; TNF, tumor necrosis factor; LTB, leukotriene; NADPH, nicotinamide adenine dinucleotide phosphate; Flii, flightless I; MIP1α, macrophage inflammatory protein1α; Trem1, Triggering receptor expressed on myeloid cells-1*.

The pro-inflammatory milieu induces the **(iii) attraction of different leukocyte populations** (Table 5). Unfortunately, the composition of these cells has not been investigated thus far in EBA patients, but it is known from BP patients that the infiltrate includes cells such as lymphocytes, histiocytes, eosinophils, neutrophils, and mast cells (228, 229). Subsequent mechanistic studies using the antibody transfer-induced model have uncovered neutrophils as the major culprits responsible for blister formation (208). The recruitment of neutrophils into the skin is mediated by CD18- and ICAM-1 (216, 230–232). In addition, CD18 crucially regulates neutrophil adhesion as an indispensable step leading to tissue damage (233).

**Table 5:** Cell lineage in the effector phase.

**Cell type**	**Function**	**References**
Neutrophils	Neutrophil depletion partially protects from EBA induction by antibody transfer	(208, 216)
NKT/γδ T cells	Depletion of NK or γδ T cells partially protects from EBA induction by antibody transfer	(225)
T_regs_	Depletion of T_regs_ worsens the clinical disease manifestation in antibody transfer-induced EBA	(224)
Keratinocytes	Upon binding of COL7 antibodies, pro-inflammatory mediators are released from keratinocytes	(209, 226)
IL-10^+^ B lineage cells	IL-10, derived from IL-10+ B cells impairs neutrophil functions and impairs clinical disease manifestation in immunization-induced EBA	(227)
Monocytes/macrophages	Monocytes/macrophages induce subpeidermal splits *ex vivo*, and depletion of all myeloid cells completely protects from EBA induction by passive antibody transfer, while selective depletion of neutrophils confers partial protection	(214, 216)

*NKT, natural killer T cells; T_regs_, regulatory T cells; IL, interleukin*.

Concerning the functional role of T cells, it was recently demonstrated that T cells can enhance neutrophil recruitment into the site of inflammation by modulating the expression of the cell surface integrin CD18 on neutrophils. Interestingly, this effect was neither mediated by CD4 nor CD8 cells, but rather γδT and NKT cells (225). Interestingly, blockade of T_reg_ led to a dramatic worsening of the clinical disease manifestation in antibody transfer-induced EBA (224).

In addition to granulocytes and lymphocytes, macrophages/monocytes may be involved in the blister formation in EBA. However, the blockade of these cells is technically difficult and does not effectively impair disease progression (142). Nevertheless, more studies are needed because macrophages/monocytes are able to produce high amounts of reactive oxygen species (ROS) after immune complex stimulation and induce *ex vivo* split formation in human skin sections (214). Furthermore, the application of high concentrations of anti-COL7 IgG has been shown to induce mast cell activation, but mast cell deficient mice develop experimental EBA just like wild type animals, indicating that mast cells do not contribute to the immune-mediated tissue injury (234–236). Concerning the role of additional cell types in inflammation, a possible role of plasma cells (227) has been discussed, but further studies are needed to unravel the cellular orchestration responsible for the lesional sites.

After extravasation from the blood into the skin, **(iv) myeloid effector cells bind to the skin-bound immune complexes** in a FcγR-dependent fashion (Table 6). In EBA the full IgG molecule of the autoantibodies, but not their corresponding F(ab)2 fragments, are pathogenic. Specifically, only the full anti-COL7 IgG elicits dermal-epidermal separation when, together with PMN, incubated on cryosections of human skin (248). Likewise, and unlike the full IgG, F(ab′)2 fragments of anti-COL7 IgG do not induce clinical EBA manifestation when injected into mice (168). The central role of these Fc-FcγR interactions for mediating skin inflammation and subepidermal blistering in experimental EBA is further supported by the complete protection of mice toward EBA induction when injected with chicken anti-mouse COL7 IgY, which is known not bind to murine complement and Fc receptors (249). In addition, the therapeutic effects observed when blocking these interactions, i.e., using soluble CD32/SM101 (231), highlights the key role of Fc-FcγR interactions in EBA pathogenesis. Furthermore, IgG glycosylation has been shown to have preventive and therapeutic effects in mouse models of chronic inflammatory diseases, including EBA (191). Further studies eluted on the differential contribution of the different FcγR (250). In mice, three different activating FcγR and one inhibitory FcγR are described: FcγRI, FcγRIII, and FcγRIV are activating FcγR, all with specific binding avidities toward IgG. The FcγRIIB is the only inhibitory FcγR (250). Of note, an increased expression of FcγRIV has been demonstrated in the skin of mice with experimental EBA (181). Subsequent functional studies identified the FcγRIV as the key mediator of tissue injury in EBA. By contrast, blockade of FcγRI, FcγRIII, or both receptors in combination had no effect on the induction of experimental EBA by antibody transfer. In FcγRIIB deficient mice enhanced blistering was observed in antibody transfer-induced EBA, as well as BP (181, 251), indicating a protective role of this FcRγ in experimental EBA. In human *ex vivo* models of BP, FcγRIIA, and FcγRIIIB contributed to the autoantibody-induced tissue damage (252). Once the neutrophils are bound to the immune complexes, a multifaceted signaling cascade is initiated (Table 6). This involves activation of the retinoid-related orphan receptor (ROR)α (230), heat shock protein (HSP)90 (241), phosphodiesterase 4 (240), phosphatidylinositol-4,5-bisphosphate 3-kinase (PI3K) β and δ (238, 239), p38, AKT, ERK1/2 (244), the spleen tyrosine kinase SYK (171, 245), and src kinases (247), as well as CARD9 (246)—which have been reviewed in detail elsewhere (253). The exact temporal and spatial order of these signaling events is currently unknown. Ultimately, the signaling cascade leads to the activation of myeloid effector cells, specifically release of ROS and proteases, both of which are required for subepidermal blistering in EBA (216, 225).

**Table 6:** Receptors and signaling in the efferent phase of EBA.

**Target**	**Function**	**References**
FcγRs	Activating FcγR promote skin inflammation in experimental EBA, while the inhibitory FcγRIIB confers protection from induction of EBA by antibody transfer	(181)
FcRn	The FcRn controls the half-live of IgG. This FcRn-deficient mice are partially protected from EBA induction by antibody transfer	(185)
CD18	CD18-deficient mice are completely protected from EBA induced by antibody transfer	(216)
CD11b	CD11b-deficient develop a more severe clinical phenotype in antibody transfer-induced EBA.	(237)
C5aR1	C5aR1-deficient mice are almost completely protected from EBA induced by antibody transfer	(202, 203)
CXCR1/2	Pharmacological CXCR1/2 inhibition prevents disease progression in immunization-induced EBA	(212)
BLT1	BLT41-deficient mice are almost completely protected from EBA induced by antibody transfer	(208)
PI3Kß	PI3Kβ-deficient mice are partially protected from EBA induction by antibody transfer, through inhibition of neutrophil activation	(238)
PI3Kδ	Pharmacological inhibition of PI3Kδ impairs induction of EBA by antibody transfer and has therapeutic effects in immunization-induced EBA	(239)
Phosphodiesterase 4	Pharmacological inhibition of PDE4 impairs induction of EBA by antibody transfer and has therapeutic effects in immunization-induced EBA	(240)
RORα	RORα-deficient mice are completely protected from EBA induced by antibody transfer	(230)
HSP90	HSP90 is involved in both loss of tolerance to COL7, as well as to antibody-induced tissue damage in experimental EBA	(241, 242)
JAK2	Pharmacological inhibition of JAK2 impairs induction of EBA by antibody transfer and has therapeutic effects in immunization-induced EBA	(243)
AKT, ERK, p38	Pharmacological inhibition of these targets impairs induction of EBA by antibody transfer (ERK, p38) or impairs subepidermal splitting *ex vivo* (all)	(244)
SYK	Pharmacological inhibition of SYK or SYK-deficient mice are completely protected from EBA induction by antibody transfer	(171, 245)
CARD9	CARD9-deficient mice are partially protected from EBA induction by antibody transfer	(246)
Src kinases	Hck, Fgr and Lyn-tripple-deficient mice are partially protected from EBA induction by antibody transfer	(247)
TREM1	See Table 5	(223)
Caspase 1	Caspase-1/11-deficient mice develop antibody transfer-induced EBA similarity to wild type littermate controls	(213)

*FcγR, Fc gamma receptor; FcRn, neonatal Fc receptor; BLT, leukotriene B4 receptor; CXCR, CXC-chemokin receptor; PI3K, Phosphatidylinositol-4,5-Bisphosphate 3-kinase; RORα, retinoid-related orphan receptor-alpha; HSP90, heat-shock protein 90; JAK2, Janus kinase 2; AKT, protein kinase B; ERK, extracellular signal-regulated kinase; SYK, spleen tyrosine kinase; CARD9, Caspase recruitment domain-containing protein 9; SRC, tyrosine-protein kinase SRC; HCK, tyrosine-protein kinase HCK; LYN, tyrosine-protein kinase LYN; TREM1, Triggering receptor expressed on myeloid cells-1*.

Recently the contribution of cytokines in EBA pathogenesis has been eluted both on the morphological, as well as the functional level (254). Because biologics targeting cytokines are already in clinical use—although, with few exceptions such as rituximab and omalizumab, for other indications (255)—we here highlight their contribution for EBA pathogenesis. Several cytokines have been shown to contribute to blister formation in experimental EBA (Table 4). IL-6 acts as a pro-inflammatory cytokine in various autoimmune diseases (256, 257). Hence, IL-6 has emerged as a potential target for the treatment of autoimmune diseases, and IL-6-targeting therapies are licensed for rheumatoid arthritis and juvenile idiopathic arthritis (256). In EBA patients and in experimental EBA models, serum IL-6 levels are elevated. Unexpectedly, mice lacking IL-6 expression show an increased clinical phenotype in experimental EBA compared with controls. Furthermore, treatment with recombinant IL-6 leads to a dose-dependent reduction of clinical disease manifestation in EBA induced by antibody transfer. Thus, in experimental EBA, IL-6 has a profound anti-inflammatory activity (209). At the molecular level, the protective effect of IL-6 is mediated by the IL-6-dependent release of IL-1ra (213). In line, IL-1R–deficient mice or wild-type mice treated with either the IL-1R antagonist Anakinra or a IL-1β function blocking antibody are also partially protected from the EBA-inducing effects of anti-COL7 IgG. Mechanistically, IL-1β increases ICAM-1 expression on endothelial cells, suggesting that IL-1β supports recruitment of inflammatory cells into the skin. Interestingly, these effects of IL-1 were independent of caspase-1 because of caspase-1/11–deficient mice showed a similar phenotype to wild type control animals when injected with anti-COL7 IgG (213).

Additionally, the evaluation of cytokines affecting neutrophil functions, such as IL-8 (CXCL1 and CXCL2 in the mouse) and GM-CSF in experimental EBA, showed an increased expression. To evaluate if the increased expression is of functional relevance these cytokines were inhibited by either antibodies of mice lacking expression of the respective cytokine(s). Treatment with allosteric CXCR1 and 2 inhibitors (DF2156A) impaired the induction of skin blistering in antibody transfer-induced EBA.

In a therapeutic setting, the administration of DF2156A improves the clinical manifestation of EBA after disease onset in immunization-induced EBA (212). For the evaluation of GM-CSF, anti-COL7 IgG was injected into GM-CSF-deficient mice or wild type mice treated with a function-blocking GM-CSF antibody. The induction of experimental EBA was impaired if the function of GM-CSF was blocked in comparison to appropriate controls. *In vitro* studies have demonstrated the requirement of GM-CSF for neutrophil recruitment from bone marrow into the blood and from the blood into the skin. Furthermore, GM-CSF preactivates neutrophils, leading to an enhancement of immune complex-induced neutrophil activation. In a therapeutic setting, the blockade of GM-CSF in mice with already established immunization-induced EBA has demonstrated beneficial therapeutic effects (177, 213). In addition to these cytokines, increased expression of TNF has been observed in experimental EBA, and prophylactic blockade of TNF and therapeutic use of etanercept in the immunization-induced EBA model impair the induction and progression of experimental EBA (214). By contrast, increased expression of CCL3/MIP1α is a mandatory cytokine for disease development (221).

## The Resolution Phase of EBA

For most autoimmune bullous diseases, the location of blisters differs over time, indicating a frequent, but unfortunately overall insufficient, healing process in the skin (Figure 5). Flightless I (Flii), an actin remodeling protein, has been shown to modulate the resolution of skin blistering in experimental models of EBA. *In vivo*, the induction of EBA leads to increased cutaneous Flii expression, resulting in impaired Claudin-1 and Claudin-4 tight junction protein expression, as well as a delay in the recovery from blistering (218, 258). Overexpression of Flii produces severe blistering post-induction of EBA, while decreased Flii reduces blister severity, elevates integrin expression, and improves COL7 production. In addition, topically applied Flii neutralizing antibodies improve the healing of blistered skin in murine EBA (218–220, 259).

An interesting experiment could show a protective effect of IL-10-positive plasma cells toward the neutrophil-dependent inflammation in EBA. After the start of skin inflammation, plasmacytosis is induced by injection of goat anti-mouse IgD serum and provides protection from skin inflammation and neutrophil infiltration for at least another 3 weeks. Suppression of EBA skin inflammation is abrogated by the co-injection of a neutralizing IL-10 receptor antibody. Despite its anti-inflammatory effect, plasmacytosis neither reduces the numbers of autoreactive COL7-specific plasma cells nor the autoantibodies that trigger the disease (227). These observations point toward a significant contribution of pathways that are involved in the resolution of cutaneous involvement. Therefore, EBA may manifest not only when many pro-inflammatory stimuli are present but also when the balance of pro-inflammatory, anti-inflammatory, and resolving pathways are unbalanced, driving the cells toward pro-inflammatory mechanisms.

CD11b is an integrin family member that pairs with CD18 to form the CR3 heterodimer. CD11b is expressed on the surface of many leukocytes, including monocytes, neutrophils, natural killer cells, granulocytes, and macrophages. Unexpectedly, in the antibody transfer-induced model of EBA, CD11b-deficient mice develop more severe disease symptoms than wild-type mice in the late phase of the disease. Furthermore, compared to wild-type controls, CD11b-deficient mice express increased levels of circulating IFN-γ and IL-4, suggesting an anti-inflammatory role for CD11b in the resolution phase of experimental autoimmune diseases, such as EBA (237).

## Emerging Treatments

The current (unsatisfactory) treatment options for EBA have been outlined above. An increasing understanding of EBA pathogenesis indicates new potential therapeutic targets that interfere in different phases of disease progression, including the generation of autoantibodies, maintaining autoantibodies in the circulation, and autoantibody-induced tissue injury.

Due to the lack of clinical studies, we categorized the emerging treatments into three categories:

## Treatments Described in Case Reports

Several case reports are known for the treatment of EBA using ECP (145) and Daclizumab (148). Furthermore, anti-C1s mAb, a mouse monoclonal IgG2a antibody, inhibits the activation of C1s, which is a serine protease of the classical complement system. TNT003 completely blocks complement classical pathway activation by the reduction of C4a and C5a production induced by incubation of the sera from patients with BP on cryosections of human skin (260). The disease induction of experimental EBA is also complement-dependent. This finding suggests that TNT003 might work even in EBA. A “humanized version” of TNT003, TNT009 is currently evaluated in a phase I clinical trial in BP patients (NCT02502903) and data reported on congresses indicates a favorable safety, as well as efficacy in some patients. Sulfasalazine was used in a patient with EBA associated with Crohn's disease, resulting in no improvement of the skin lesion (149). The utility of doxycycline has been reported in BP (150). Doxycycline and another tetracycline, minocycline, have been described in the literature in EBA cases, although their usefulness remains unclear (151–153).

## Treatments Applied in Therapeutic Settings in Experimental EBA

Due to the low incidence, clinical studies with EBA patients are not performed. Therefore, animal models are indispensable for investigating the pathogenesis of EBA and other autoimmune blistering diseases and to test new target substances for treatment; these models include the transfer of autoantibodies to experimental animals, adoptive transfer of autoantibody-producing B cells to immune-deficient mice and construction of transgenic mice that produce autoantibodies (167). Although animal models have many benefits, as described above, both the advantages and disadvantages of each model should be considered. Although the models duplicate the clinical, histopathological, ultrastructural, and immunological features of the human disease, these systems are mostly completely murine, and it is possible that findings in mice will not translate to humans.

The most important mouse model to test treatments applied in therapeutic settings is the immunization-induced EBA model (167, 168). Immunization with parts of the NC1 domain of COL7 with an adjuvant induces anti-COL7 IgG production in mice. The susceptibility of experimental EBA is closely associated with the H2s haplotype in mice (165). During a time period of 4–6 weeks, the mice develop antibodies against mCOL7 that bind to the DEJ and induce neutrophil activation and blistering. Using the immunization-induced EBA model, the pathomechanisms of blistering and potential therapeutic options have been studied, and more importantly, this model is used to test potential new drug candidates and therapeutics (167). Successfully applied drugs are summarized in Table 7.

**Table 7:** Experimental treatments in pre-clinical immunization-induced EBA.

**Medication**	**Target**	**Impact on disease**	**Application**	**References**
Methylprednisolone	Multiple	Impair EBA progression	i.p.	(129, 212)
DF2156A	CXCR1/2	Halting EBA progression	p.o.	(212)
IVIG	Multiple	Impair EBA progression	i.p.	(129)
Anti-GM-CSF	GM-CSF	Impair EBA progression	i.p.	(177)
Etanercept	TNFα	Impair EBA progression	i.p.	(214)
EndoS	Fc glycosylation	Halting EBA progression	i.p.	(192)
sCD32/SM101	FcγR	Impair EBA progression	i.p	(231)
Anakinra	IL-1	Improvement	i.p.	(213)
17-DMAG	HSP90	Improvement	i.p.	(174)
17-AAG	HSP90	Impair EBA progression	top	(242)
TCBL-145	HSP90	Improvement	i.p.	(174)
Dimethylfumarate	Multiple	Improvement	p.o.	(261)
goat anti-mouse IgD serum	Induction of IL10 plasma cells	Impair EBA progression	i.p.	(227)
Calcitriol treatment	Vitamin D	Impair EBA progression	p.o.	(262)
LAS191954	PI3Kδ	Improvement	p.o.	(239)
Roflumilast	PDE4	Halting EBA progression	p.o.	(240)

*CXCR1/2, CXC-chemokin receptor; GM-CSF; granulocyte-macrophage colony-stimulating factor; TNF, tumor necrosis factor; EndoS, endoglycosidase S; FcγR, Fc gamma receptor; IL, interleukin; 17-DMAG, 17-dimethylaminoethylamino-17-demethoxygeldanamycin; HSP90, heat shock protein 90; 17-AAG, Tanespimycin (17-N-allylamino-17-demethoxygeldanamycin); TCBL-145, D-Tyr-Phe-D-Trp-Leu-AMB (AMB: NH-CH_2_-CH_2_-CH(CH_3_)_2_); PI3K, phosphoinositide 3-kinase; p.o., per os; i.p., intraperitoneal; top, topical*.

## Treatments Applied in Preventive Settings in Experimental EBA

Beneath the validation of potential drug candidates in the therapeutic setting in immunization-induced EBA, some authors have also investigated several drugs in antibody transfer-induced EBA. In this model, rabbit sera or whole rabbit IgG immunized with COL7 NC1 domain are injected into mice. These mice develop clinical and histological subepidermal blistering within a few days. The antibody transfer models are used to investigate the cellular and molecular basis of blistering and to investigate preventive effects of several therapeutic options. The use of antibody transfer models enables examination of the effector phase of EBA. Testing novel drugs or investigating the pathogenesis of EBA is straightforwardly done because clinical symptoms are visible days after starting the experiment. However, similar to most animal models, the situation in human patients is only partially reflected—mostly, because the signaling cascade and the interaction of different cell types vary among human and mice. Additionally, induced disease may last less than a few days after the transfer of antibodies. Furthermore, the loss of tolerance and the generation of autoantibodies cannot be studied in models based on antibody transfer. Therefore, the investigation of potential drug candidates should always be performed in addition to immunization-induced EBA in a therapeutic setting.

### Complement

Complement activation has been described as a prerequisite for blister formation. In the antibody-transfer model, blister induction is completely dependent on complement C5 because C5-deficient mice or mice injected with F(ab)2 fragments of the immune IgGs are devoid of blisters. This finding indicates that the complement system can be targeted in animal models. Targeting therapies for complement system factor B, C5, and C5R ameliorate disease severity in antibody-transfer models (199, 201, 203, 211, 260). Interestingly, in antibody transfer-induced BP, C5ar1^(−/−)^ mice are protected from disease development, whereas the extent of skin lesions is increased in C5ar2^(−/−)^ animals.

### SYK

SYK is a non-receptor cytoplasmic enzyme that is mainly expressed in hematopoietic cells. SYK regulates cellular responses to extracellular antigens or antigen-immunoglobulin complexes. In EBA pathogenesis, myeloid SYK is a central player in driving inflammation in prototypical autoantibody-transfer models. The SYK inhibitor (BAY61-3606) protects mice from inflammation in antibody-transfer EBA (171, 245).

### Cell-Derived Nanoparticles (CDNPs)

CDNPs are intercellular protein complexes, such as annexin A1, annexin A5, actin, *14-3-3*ε*, 14-3-3*ζ, galectin-3, and heat-shock proteins 27 and 70. CDNPs play a therapeutic role against viral infections, cancer and in the experimental EBA model. Mice that receive anti-COL7 IgG develop skin lesions. Treatment with CDNPs significantly reduces the affected skin lesions and increases IL-4 expression (263).

### LTB4

LTB4 is a potent chemoattractant and activator of myeloid cells, particularly of neutrophils. The 5-lipoxygenase–deficient and BLT1-deficient mice exhibit neither any clinical nor histological signs of disease in the antibody-transfer model. The 5-lipoxygenase inhibitor (zileuton) targeting the LTB4/BLT1 pathway ameliorates disease severity by ~50% in antibody-transfer models (208).

### Flii I

Flii is a member of the gelsolin family of actin remodeling proteins in regulating cell adhesion and intracellular signaling. Flii expression is increased in the epidermis during development and during epidermal stratification to maintain barrier functions, such as tight junctions. Topical application of Flii-neutralizing antibody significantly reduces the clinical severity of blistering and histological separations in antibody-transfer EBA mice. In addition, the barrier function measured by transepidermal water loss (TEWL) is significantly decreased in Flii-neutralizing antibody-treated mice (218, 219).

### IL-6 (Protective)

IL-6 is a proinflammatory cytokine and plays a role during the transition from innate to acquired immunity. In the initial immune response to pathogen, IL-6 attracts neutrophils to the affected tissue. In addition, IL-6 plays a crucial role in B- and T-cell differentiation. Elevated IL-6 concentrations are observed in many inflammatory diseases and often correlate with disease activity. IL-6 expression is increased in the skin of EBA patients. Interestingly, the EBA severity is enhanced in IL-6-deficient mice in an experimental model. Furthermore, treatment with recombinant IL-6 dose-dependently impairs the induction of experimental EBA (209).

### Galactosylated Immunoglobulins

Antibody-transfer EBA model activation is FcgR-dependent in blister formation. Immune-complexes induce the classical complement pathway via Fc fragment. The mouse IgG1 subclass preferentially binds to inhibitory FcgRIIB and suppresses the inflammatory response. Glycosylation of Asn297 in the IgG Fc fragment plays an important role in complement activation. Highly galactosylated immune-complex treatment reduces the development of cutaneous lesions in an antibody-transfer model (202).

### CARD9

Caspase recruitment domain-containing protein 9 (CARD9) is an intracellular adapter protein that is expressed in myeloid-lineage cells. CARD9 plays a critical role in host defense against pathogens in both mice and humans. Card9 deficient mice are significantly protected against skin blistering in antibody-transfer EBA. In this setting, CARD9 is required for development of the inflammatory response. These pieces of evidence suggest a therapeutic target in EBA (246).

### Signal Transduction by Scr Kinases

Src family kinases, such as Hck, Fgr, and Lyn, play roles in malignant transformation and tumor progression, and therefore they can be targets of cancer therapy. In addition, Src family kinases are present in many types of immune cells. It is known that Src family kinases have a role in integrin signal transduction in neutrophils and macrophages. Hck, Fgr, and Lyn-deficient mice are completely protected in antibody-transfer EBA (247).

### T Cells

T_regs_ are of importance in modulating host responses to tumors and infections and in inhibiting the development of autoimmunity and allergies. T cell-deficient mice are protected from induction of skin lesions in antibody-transfer EBA. In particular, specific depletion of T_regs_ increases disease progression in antibody transfer-induced EBA. In a similar experimental setting, NKT-deficient mice and γδT cell-deficient mice are protected against the induction of experimental EBA (224, 225).

### Signal Transduction by p38, MAPK AKT

In the antibody-transfer model, neutrophils are crucial for inducing clinical disease. Neutrophil activation is mediated by the phosphorylation of ERK1/2, p38 MAPK, and Akt. Methylprednisolone inhibits the phosphorylation of Akt, ERK1/2, and p38 MAPKs in neutrophils. Chemical inhibitors of Akt (Akt inhibitor VIII), ERK1/2 (U0126), and p38 MAPK (SB203580) statistically suppress *ex-vivo* dermal-epidermal separation. In addition, ERK1/2 (U0126), and p38 MAPK (SB203580) demonstrate an ~10% reduction of disease severity compared with the control (244).

### RORα

RORα is a steroid nuclear hormone receptor and a transcription factor. Experimental EBA shows strain-dependent disease severity. To elucidate the strain-dependency, RORα is found to be a risk gene for the antibody-transfer model. The RORα agonist SR3335 impairs blister formation in both types of antibody-transfer EBA (230).

### Signal Transduction by PI3Kβ

PI3Ks play major roles in the signaling pathways that link cell surface receptors to the control of cell function. There are four defined isoforms of PI3Ks. In particular, PI3Kβ is widely expressed and blocks arterial thrombus formation. PI3Kβ-deficient mice are protected from the development of blisters in the antibody-transfer EBA model.

### Others

This antibody-transfer mouse model can be used to validate or, at least as important exclude novel therapeutic targets in EBA. For example, TREM1, MIP1a, CD11b, caspase 1/11, and S100, which are all increasingly expressed in experimental EBA, have no impact on disease manifestation (213, 221–223, 237).

## Conclusions

Since the first description of EBA over 100 years ago, our understanding of this serious disease has dramatically improved. The knowledge of the cellular and molecular mechanisms led to the development of new therapies, some of which have been successfully tested in preclinical models and in concept studies in bullous pemphigoid patients. In the future, new drug targets will evolve to improve the treatment and living standards of people with EBA.

## Author Contributions

All authors listed have made a substantial, direct and intellectual contribution to the work, and approved it for publication.

### Conflict of Interest Statement

The authors declare that the research was conducted in the absence of any commercial or financial relationships that could be construed as a potential conflict of interest.

## Abbreviations

17-AAG: tanespimycin
17-DMAG: 17-imethylaminoethylamino-17-demethoxygeldanamycin
ABQOL: autoimmune bullous disease quality of life
AIBD: autoimmune blistering disease
AKT: protein kinase B
APC: antigen-presenting cell
AZA: azathioprine
BLT: leukotriene B4 receptor
BMZ: basement membrane zone
BP: bullous pemphigoid
C: complement factor
CARD9: caspase recruitment domain-containing protein 9
CD: cluster of differentiation
CDNP: cell-derived nanoparticles
COL: collagen
CPA: cyclophosphamide
CR: complete remission
CSA: cyclosporine
CXCR: CXC-chemokin receptor
DDS: diaminodiphenyl sulfone
DEJ: dermal-epidermal junction
DIF: direct immunofluorescence
DMF: dimethylfumarate
EBA: epidermolysis bullosa acquisita
ECP: extracorporeal photochemotherapy
ELISA: enzyme-linked immunosorbent assay
EndoS: endoglycosidase S
ERK: extracellular signal-regulated kinase
FcgR: Fc gamma receptor
FcRn: neonatal Fc receptor
Flii: flightless I
FOAM: fluorescent overlay antigen mapping
G0: agalactosylated antibodies
GM-CSF: granulocyte-macrophage colony-stimulating factor
HLA: human leukocyte antigen
Hsp: heat-shock protein
i.p.: intraperitoneal
IA: immunoadsorption
IC: immune complexes
IEM: immunoelectron microscopy
IFN: interferon
Ig: immunoglobulin
IIF: indirect immunofluorescence microscopy
IL: interleukin
IVIG: high-dose intravenous immunoglobulin
JAK2: janus kinase 2
LAD: linear IgA bullous disease
LTB4: leukotriene B4
MHC: major histocompatibility complex
MIP1a: macrophage inflammatory protein1a
MM-EBA: mucous membrane EBA
MMF: mycophenolate mofetil
MMP: mucous membrane pemphigoid
MMPs: matrix metalloproteases
MTX: methotrexate
NADPH: nicotinamide adenine dinucleotide phosphate
NC: non-collagenous
NCF1: neutrophil cytosolic factor 1
NKT: natural killer T cells
p.o.: per os
PI3K: phosphatidylinositol-4,5-bisphosphate 3-kinase
PR: partial remission
RORa: retinoid-related orphan receptor-alpha
ROS: reactive oxygen species
RTX: rituximab
SLE: systemic lupus erythematodes
SSS: NaCl-split skin
SYK: Spleen tyrosine kinase
TABQOL: treatment-based autoimmune bullous disease quality of life
Th: T-helper
TNF-α: tumor necrosis factor α
Treg: regulatory T cells
Trem1: triggering receptor expressed on myeloid cells-1.
